# Essential information for transition of care for frail elderly patients in Japan: A qualitative study

**DOI:** 10.1002/jgf2.478

**Published:** 2021-07-09

**Authors:** Shinji Matsumura, Makiko Ozaki, Tetsuya Kanno, Tomomi Iioka, Seiji Bito

**Affiliations:** ^1^ Department of Clinical Epidemiology National Hospital Organization Tokyo Medical Center Tokyo Japan; ^2^ Matsumura Clinic Tokyo Japan; ^3^ Internal Medicine Murasakino Kyoritsu Clinic Kyoto Japan; ^4^ Marufuku Home Clinic Tokyo Japan

**Keywords:** elderly, geriatrics, Japan, qualitative research, referral letter, transition of care

## Abstract

**Background:**

Information exchange between hospitals and primary care physicians is suboptimal. Most physicians are dissatisfied with the current referral process, and poor communication leads to negative care transition outcomes.

**Method:**

To identify the key information needed for a successful transition of care, we conducted a qualitative study using consecutive, semistructured in‐person interviews and focus group sessions. We recruited five participants engaged in clinical work for individual interviews and 16 participants for focus groups. We analyzed all data using qualitative thematic analysis. All results were returned to the participants and modified based on their feedback.

**Results:**

The five individual interviews provided a general picture of the current referral process and an interview guide for the following focus group sessions. The focus group discussions were used to identify the essential information needed at admission and discharge from the hospital. Essential information on hospital admission was as follows: (1) basic medical and care information, (2) care resources available at home, (3) the purpose of admission and the goals of care during hospitalization, and (4) status of advance care planning (ACP) and patient's will in an emergency. Essential information on hospital discharge was as follows: (1) clinical course, (2) explanation of medical condition during hospitalization, (3) status of ACP and patient's will in an emergency, and (4) medical procedures to be continued at home.

**Conclusions:**

We identified the essential information needed for a successful transition of care in Japan. The clinical effectiveness of a template that contains the information identified in our study warrants further investigation.

## Abstract 2

To identify the key information needed in referral letters for a successful transition of care, we conducted a qualitative study using consecutive, semi‐structured in‐person interviews and focus group sessions. Essential information on hospital admission and hospital discharge was identified. We identified essential information needed for the clinical effectiveness of a template that contains the information identified in our study warrants investigation.

## 1 BACKGROUND

Changes to population demographics challenge health systems around the world, with elderly people accounting for the largest increase in hospital admissions and discharges.^1^ As the world's population ages, clinical complexity increases, as does the prevalence of comorbidity, functional disability, and social complexity.^2^ In particular, social complexity places a massive burden on healthcare systems in terms of medical expenditure.^3^, ^4^, ^5^

The burden on cost containment leads to shorter periods of hospital stay. This is reflected worldwide, with the duration of hospitalization in Japan having dropped dramatically from 24.8 days in 2000 to 16.2 days in 2018.^6^ This can lead to shorter periods of transition. There is therefore growing interest in transitional care interventions that promote the safe and timely transition of patients between levels of care and across settings, such as between hospitals and primary care physicians.^7^, ^8^, ^9^, ^10^, ^11^, ^12^

However, studies to date have shown that information exchange between hospital and primary care physicians is suboptimal. Studies demonstrate that most physicians, both primary care and hospital physicians, are dissatisfied with the current referral process, the contents of referral letters, and the lack of information on items such as current medication, prior treatment, and continued treatment.^11^, ^13^, ^14^, ^15^, ^16^ Poor communication between physicians leads to negative care transition outcomes, such as discontinuity of care, compromised patient safety, dissatisfaction by patients and caregivers, and ineffective use of health resources.^11^, ^13^, ^17^, ^18^, ^19^

To date, many interventions have been performed to assess and improve the quality of the referral process. One approach to improve the quality of referral letters is to use a template that ensures that necessary information is provided in a concise manner.^20^, ^21^, ^22^ However, few studies have been conducted to identify the essential information, especially in the transition of care for frail elderly patients.

The purpose of this study was to identify the key information needed for inclusion in referral letters for successful transition of care. In particular, we aimed to identify the key information needed for successful acute hospitalization of frail elderly patients with complex social needs.

## 2 METHODS

In this qualitative study, we conducted consecutive, semi‐structured in‐person interviews with five participants and two focus group (FG) sessions with 16 participants who were engaged in clinical work from January to March 2016. First, we conducted qualitative thematic analysis of the semi‐structured interviews to explore the basic assumptions related to the current referral process in care transition in Japan. We then conducted the FG sessions, which comprised discussions around specific themes related to vital information needed for care transition. The interview guide consisted of a series of open‐ended questions created for this study by the researchers (see Additional file 1).

SM and MO are primary care physicians and health service researchers with a background in qualitative analysis. TI is a pharmacist with a background in qualitative analysis. SM, MO, and TI jointly participated in data collection and analysis.

The Institutional Review Board at the National Hospital Organization Tokyo Medical Center reviewed and approved the study and procedures (application number: R15‐111).

### 2.1 Subjects and settings

All participants of the individual interviews were invited by the investigators with emails to participate through their clinical networks. The participants comprised a visiting nurse, a primary care physician in a clinic, a primary care physician working at a small community hospital, a case manager at an acute hospital, and a community care manager.

Focus group sessions were conducted in an interview room located in the Tokyo metropolitan area. We used convenient sampling to recruit participants but tried to include as diverse a group in terms of age, gender, specialty, and workplace as possible. The first session aimed to explore the perspectives of acute hospital workers; thus, five physicians, two nurses, and one medical social worker in an acute hospital were recruited. The second session aimed to explore the perspectives of primary care workers; thus, three primary care physicians, one community pharmacist, one visiting nurse, one nurse at a nursing home, and two community care managers were recruited (Table 1). All participants were employed at different workplaces.

**TABLE 1:** Professions of focus group participants

	Session 1 (8 participants) Acute hospital staff	Session 2 (8 participants) Community care staff
Profession	Physician (general internal medicine/acute hospital) Physician (community medicine/teaching hospital) Physician (emergency medicine/acute hospital) Physician (emergency medicine/acute hospital) Physician (general internal medicine/acute hospital) Nurse (discharge support center/acute hospital) Nurse (discharge support center/acute hospital) Medical social worker (community association section/acute hospital)	Physician (family physician/clinic) Physician (family physician/clinic) Physician (general Internist/home care clinic) Pharmacist (community pharmacy) Nurse (visiting nurse) Nurse (nursing home) Care manager (home care support services in the community) Care manager (community comprehensive care center)

### 2.2 Data collection

SM conducted all in‐person interviews, and MO, TI, TK, and SM jointly conducted the FGs. Each FG was led by one facilitator and one cofacilitator. Individual interviews were conducted for approximately 30  min, and the FGs, for approximately 2  h.

The topics discussed in the individual interviews were (1) important clinical information at admission for frail elderly patients, (2) important clinical information at discharge, (3) potential benefits of early information transfer both at admission and at discharge, and (4) desirable format for smooth and timely transfer of essential information.

In the FGs, we used a moderator guide to progress through topics derived from the individual interviews and discussion among the researchers.

### 2.3 Data analysis

All interviews were audio‐recorded, anonymized, and transcribed verbatim. We analyzed all data using thematic analysis. Interview transcripts were analyzed inductively and reflectively. First, two researchers (MO and TI) independently and repeatedly read through all the data and coded them according to meaning chunks. They subsequently grouped similar codes into subcategories to provide insight into meaningful topics and codes. Finally, all results were merged and reconciled through repeated discussion among the researchers (MO, TI, and SM). All of the results were then returned to the participants, who provided feedback, which the researchers incorporated into the results. To ensure trustworthiness, we validated all our findings by presenting them to the patients of a self‐help group who had recently been discharged from acute hospitals for comments and suggestions.

## 3 RESULTS

**FIGURE 1:**
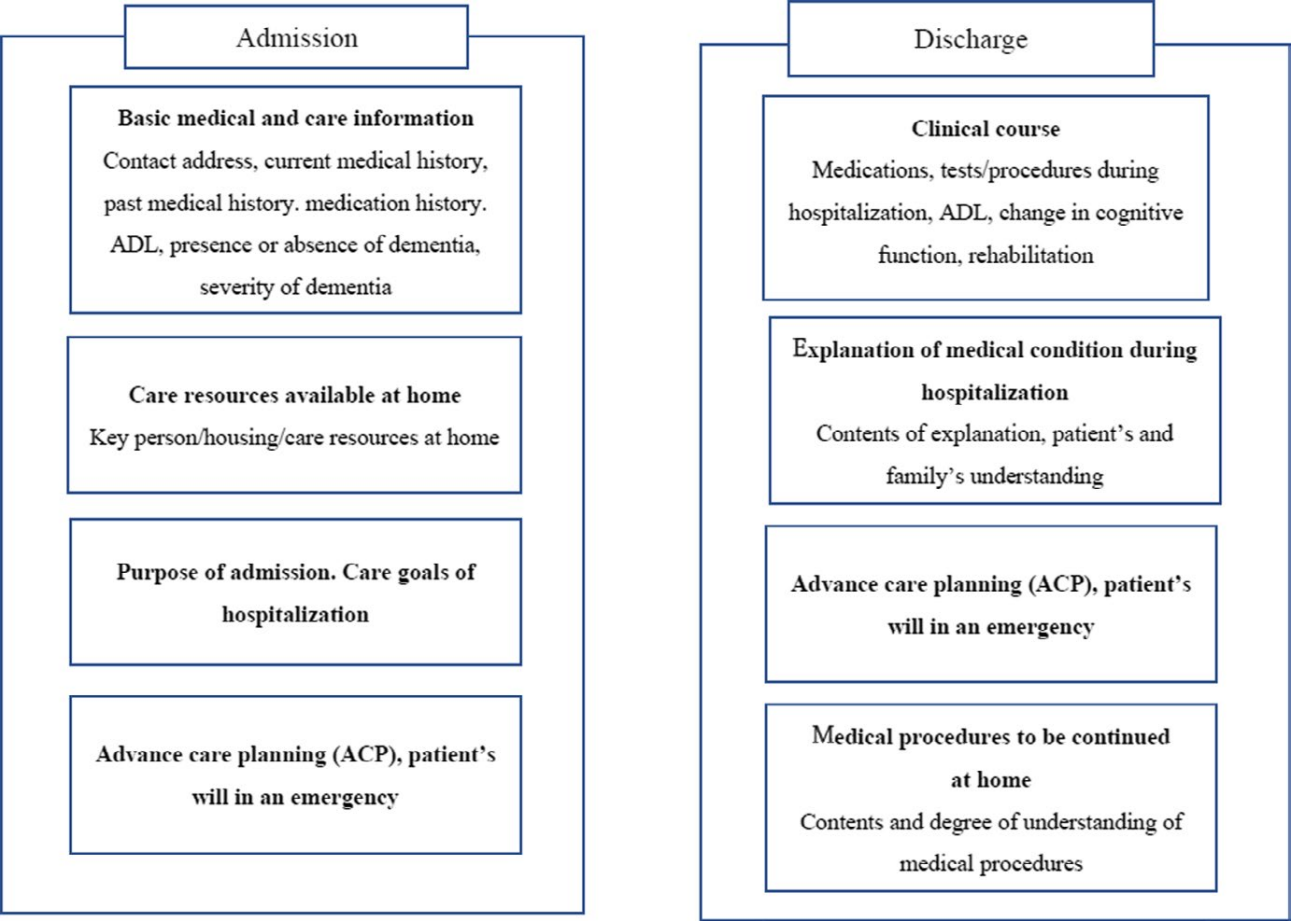
Information needed at care transition, on hospital admission/discharge

The five in‐person interviews provided a general picture of the current referral process and a useful interview guide for the following FG sessions. Briefly, all participants admitted that they experienced problems in care transition, and all agreed to develop a template with the necessary information for admission to and discharge from hospital for frail elderly patients, especially home‐bound elderly patients with multiple care needs. All participants agreed that a different set of information is needed for care transition on admission compared with discharge. Figure 1 illustrates FG participants' views about essential information needed for care transition in Japan. We present these results separately below.

(1) **Essential information on hospital admission**

### 3.1 Basic medical and care information

Participants remarked that basic medical and care information is crucial. Examples of such information are the patient's or a family member's contact information and the patient's past medical history, current activities of daily living (ADL), and medication history. Most of this information is generally already provided in referral letters, but a template may be useful and would improve the ease with which health care staff can find such information.

One participant said,

I think past medical history, medication, and family information or ADL of patients are usually written in referral letters. But we are very grateful when all this information is listed in one place. (physician, acute hospital)

Care information is also very important.

We have a database containing all of the care users' information…and based on that, we can provide basic care information for home care, but we are weaker in (collecting) medical information. For example, ADL is usually not listed in such a database, so we need to send this information to the admitted hospital separately. So, if it's an emergency admission, we will usually send a referral later when the hospital needs it. (care manager, community comprehensive care center)

### 3.2 Care resources available at home

The participants also identified details related to care resources, housing, and key family members for decision making as important information for admission.

… It is really great when (the letter) contains details about care resources and decision‐making capacity, especially regarding resuscitation. And information on the key people in the family is really, really important. (physician, acute hospital)

Regarding the contents (of the referral letter), we actually don't need the clinical information in that much detail. Instead, some primary care physicians note the key people for the patient and their understanding of the patient's current status. I really appreciate that. (nurse, acute hospital)

### 3.3 Purpose of admission and care goals of hospitalization

Participants of the FGs suggested that the purpose and goal of hospitalization must be written in the referral letter. Further, participants from the community remarked that the information should include procedures that can be performed at home to enable them to easily assess the care/treatment goals during hospitalization.

For example, the referral letter from the home‐care team should contain information about the expected goal of the treatment. (nurse, community)

For example, when the family does not want the patient to receive curative treatment, but just wants pain relief. It is rare, but we really appreciate it when we read a letter that includes a statement saying that the home‐care team can provide such palliative care at the patient's home. It is great. (physician, acute hospital)

### 3.4 Status of advance care planning (ACP) and patient's will in an emergency

Many participants, especially those from acute hospitals, discussed the importance of details related to the status of ACP. For patients who have already discussed their preference for care at the end of life, this information should be provided as a top priority. However, most of the time, such information is missing from the referral letter.

One physician at an acute hospital said,

If the patient had discussed advance care planning details while at home, and the referral letter includes that information, I think the treatment for rehabilitation may differ. Most referral letters do not include (such information). (physician, acute hospital)

Another community physician said,

As a physician providing home‐visit care, I always emphasize continuity at care transition. Most of the patients that I see now cannot visit the hospital or are patients with terminal cancer. So, I think I need to pay attention to what the patient wants to do for the rest of his/her life. So, I would like to transfer such information (to the hospital), including the kind of care they want to receive and the kind of care they do NOT want to receive. (physician, community)

(2) **Essential information on hospital discharge**

### 3.5 Clinical course

Participants expressed that a summary of the patient's clinical course is essential, including medications, past medical history, test findings, and procedures received during hospitalization. In particular, information about changes in medicine is often lacking. Additionally, despite being very important for the care transition process, information on ADL and the patient's cognitive status during hospitalization is often missing.

(Medication information), for both internal and external medicine, is often missing. If a patient is hospitalized in the internal medicine ward, we sometimes think it is unnecessary to write the name of the patient's eye drops in the hospital chart. (physician, acute hospital)

"For example, diet, toileting, consciousness, physical restraint, paralysis, speech, wounds… if a patient is discharged to the care facility in xx community, we always write all of these details down (in the letter). (physician, acute hospital)

### 3.6 Explanation of medical condition during hospitalization

Focus groups with community staff, in particular, explored the need to provide an explanation of patients' medical conditions during hospitalization.

Yes, definitely we would like to know how the physician in the hospital explained his/her medical condition. Often, it is simply one sentence, like ‘he or she improved, so he/she can return home.'… But, if that's all they heard, the description depends on what they heard about how their condition improved at all. I always want to ask about that to confirm the patient's will in the future. (nurse, community)

An additional important column, as I mentioned earlier, containing a description of the disease, prognosis, informed consent, and so on. I'd like the doctor to write a letter that includes all of this information. (physician, acute hospital)

### 3.7 Status of ACP and patient's will in an emergency

If APC was processed during hospitalization, such information should be provided to staff in the community after discharge.

Even though the prognosis of cancer or COPD is determined using a framework of the terminal stage of the disease, there are still family members who call an ambulance (after discharge) because of the pain the patient is experiencing. Even in such cases, we can contact them as soon as the prognosis has been determined (in the hospital)… (physician, acute hospital)

### 3.8 Medical procedures to be continued at home

Participants in the community, in particular, indicated the importance of providing information about necessary medical treatment or procedures to be continued at home, the details of these procedures, how the patient and caregivers are taught, and the level of patient/caregivers' understanding.

Nutrition, such as diet, is critical. And the medical treatment that directly affects life is also important. And to what extent the patient or caregiver can provide this information is critical, I think. But this is difficult to discern from only documents. (nurse, community)

### 3.9 Strengths and weaknesses of smooth information transfer

Focus group participants discussed the strengths of smooth information transfer using a referral template. From the healthcare staffs' perspective, using a template will have a positive impact on the continuity of care, reduce information duplication, and enhance care efficiency. It may also improve the quality of care, such as safety and patient satisfaction.

It is not about an only hospital or only home care… but basically, both physicians and nurses together provide care and share the goal… (physician, acute hospital)

… Maybe this will be the merit for patients and families. We don't need to repeat the same story again and again… that is definitely a merit (everybody agrees). (physician, community)

And, with regards to ‘a conflict’, it may reduce conflict between doctors, between patients and doctors. It also saves time. (physician, acute hospital)

### 3.10 Format of the referral letter

The participants of the FGs agreed that a set format for information transfer is necessary and useful but had different opinions on the actual format.

It seems like it will be easy to write the letter if clinical and other information is separated, and we can write freely. If we have lots to write, such as the profile of the patient's family, and the letter is named ‘clinical information referral letter’ (Shinryo‐joho‐teikyo‐syo), it may be difficult to write such information… So, maybe the letter itself should be renamed; for instance, ‘Family member information letter’ or ‘Supplement letter,’ etc. If the name is different… then we can write whatever we want, as much as necessary. (physician, acute hospital)

If we have a common tool to help us write the letter, it would be appreciated. And I hope it will be possible to exchange letters in a common format. And if we could send it online, it would be even better. (nurse, community)

## 4 DISCUSSION

The present study provides key insights into information exchange during care transition for frail elderly patients in Japan. To our knowledge, this is the first study to collect the opinions of staff who are currently engaged in care transition and reflect their actual experiences in Japan.

The main findings of this study are the differences in the information needed on hospital admission and discharge. On admission, basic medical and care information, care resources available at home, the purpose of admission and goals of therapy, and details of ACP are important. On discharge, clinical course, explanation of medical condition during hospitalization, ACP, and necessary medical interventions were considered important. The participants preferred a simple, paper‐based template such as a single A4 page but suggested that Web‐based information platforms may be promising in the future.

Our research describes a number of important findings that are relevant to our current transitional process in Japan. First, our study revealed that the current process of information transfer is suboptimal. Information lacking in the care transition process identified in previous studies includes prior investigations,^23^ medication,^24^ and adequate attention to assessing comprehensive social conditions, especially among the elderly.^25^ Our participants identified similar items, and all agreed that improving information transfer may improve the current care transition process.

Second, we found that some key information identified as being necessary on hospital admission overlapped with those on discharge; however, they differ by several important points. On admission, the patient's and caregiver's care information, housing, and care resources at home were considered important. On discharge from the hospital, information related to the patient's time in the hospital, such as clinical course, explanation of their medical condition, and necessary procedures after discharge, was key considerations.

Third, all participants agreed that both on admission and on discharge, the ACP process's status should be securely transferred. While the ACP process should be initiated while the patient is at home, reports have shown that most predetermined preferences fail to be conveyed when the patient is in the emergency department or being discharged.^26^, ^27^ Transition of care may provide a good chance to confirm the status of the patient's ACP process.^28^ Our findings support these perspectives. Because the re‐admission rate is about 10% within 30 days among the elderly,^29^, ^30^, ^31^ this ACP information should be securely shared at discharge.

Finally, most participants agreed that the format should be concise and paper‐based, although Web‐based information transfer is expected in the future. Such a Web‐based approach has already been discussed in Japan,^31^ indicating the need to create systems for secure, Web‐based, electronic databases that contain patient's clinical information.

Several limitations warrant mention. First, our recruitment process likely led to the selection of healthcare providers in the Tokyo metropolitan area, where health resources are relatively affluent. Therefore, our findings may not be generalizable to other areas, such as rural areas or areas where healthcare resources are scarce. Second, while we tried to recruit participants with as diverse backgrounds as possible, there was little difference in their opinions. Different thoughts or views may be more evident if we include additional professionals, such as home helpers or care staff. Third, because half of the participants were physicians, our information may be skewed to their perspective. Although the purpose of the current study was to identify information needed at care transition from the perspective of medical professionals, particularly at admission to and discharge from acute hospitals, the findings may differ for care transition at postacute facilities, such as rehabilitation centers or long‐term care facilities. However, because hospital admission and discharge account for the majority of care transition cases in Japan, collecting data from medical professionals is justified.

## 5 CONCLUSION

We identified the essential information needed for care transition in Japan, particularly on admission to and discharge from acute hospitals. Further study is needed to examine the clinical effectiveness of a template that contains the information identified in our study.

## CONFLICT OF INTEREST

The authors have stated explicitly that there are no conflicts of interest in connection with this article.

## Supporting information

Additional file 1
