# Supplementary material for: Essential information for transition of care for frail elderly patients in Japan: A qualitative study
Source: J Gen Fam Med. 2021 Jul 9;23(1):24–30. doi: 10.1002/jgf2.478 (PMC8721333; doi:10.1002/jgf2.478)
Supplement: Supplementary file 1 — Additional file 1 [file JGF2-23-24-s001.docx]

**Interview Guide**

1) Participant Background

Profession, job description, working environment, years of clinical experience.

2) What medical and care information might be needed for a frail elderly patient to be hospitalized?

　What is the essential information in the referral letter?

　What care and living information should be necessary immediately after admission?

　What is the problem with not having such information and what are the challenges you are currently experiencing?

3) What medical and care information might be needed for a frail elderly patient at the time of discharge?

　What is the essential information in the referral letter?

What care and living information should be necessary immediately after discharge?

What is the problem with not having such information and what are the challenges you are currently experiencing?

4) What are the potential benefits of early information transfer to the patient/medical staff at admission and discharge? What is the desirable format for smooth and timely transfer of information?
